# Mechatronics and Remote Driving Control of the Drive-by-Wire for a Go Kart †

**DOI:** 10.3390/s20041216

**Published:** 2020-02-23

**Authors:** Chien-Hsun Wu, Wei-Chen Lin, Kun-Sheng Wang

**Affiliations:** Department of Vehicle Engineering, National Formosa University, Yunlin 63201, Taiwan; 10758102@gm.nfu.edu.tw (W.-C.L.); 40528104@gm.nfu.edu.tw (K.-S.W.)

**Keywords:** vehicle control unit, go kart, machine vision, WiFi, drive-by-wire, remote control

## Abstract

This research mainly aims at the construction of the novel acceleration pedal, the brake pedal and the steering system by mechanical designs and mechatronics technologies, an approach of which is rarely seen in Taiwan. Three highlights can be addressed: 1. The original steering parts were removed with the fault tolerance design being implemented so that the basic steering function can still remain in case of the function failure of the control system. 2. A larger steering angle of the front wheels in response to a specific rotated angle of the steering wheel is devised when cornering or parking at low speed in interest of drivability, while a smaller one is designed at high speed in favor of driving stability. 3. The operating patterns of the throttle, brake, and steering wheel can be customized in accordance with various driving environments and drivers’ requirements using the self-developed software. The implementation of a steer-by-wire system in the remote driving control for a go kart is described in this study. The mechatronic system is designed in order to support the conversion from human driving to autonomous driving for the go kart in the future. The go kart, using machine vision, is wirelessly controlled in the WiFi frequency bands. The steer-by-wire system was initially modeled as a standalone system for one wheel and subsequently developed into its complete form, including front wheel steering components, acceleration components, brake components, a microcontroller, drive circuit and digital to analog converter. The control output section delivers the commands to the subsystem controllers, relays and converters. The remote driving control of the go kart is activated when proper commands are sent by the vehicle control unit (VCU). All simulation and experiment results demonstrated that the control strategies of duel motors and the VCU control were successfully optimized. The feasibility study and performance evaluation of Taiwan’s go karts will be conducted as an extension of this study in the near future.

## 1. Introduction

A technology known as drive-by-wire, also called “x-by-wire” or “by-wire,” may change the way people drive or the service model. A vehicle with this type of system focuses mainly on electronics to control a wide range of vehicle operations, including: acceleration, braking and steering [1]. By replacing conventional throttle systems, drive-by-wire systems can significantly reduce the number of mechanical parts in a vehicle. This reduces weight and increases operational accuracy, and prolongs the period between each routine service for mechanical maintenance and other adjustments. Some drive-by-wire systems even require nearly no service. Less weight and better accuracy can lead to higher fuel efficiency and lower emissions [2].

With the growth of electronics technologies and electrical parts in vehicles, the number of vehicles equipped with drive-by-wire systems are increasing. They are highly computer-based and connected wirelessly to services such as OnStar or Toyota Safety Connect. These features enhance vehicle safety and reliability. Furthermore, a decision-making control algorithm is utilized to manage the autonomous maneuvering of the vehicle in emergency situations [3]. Kalinowski et al. developed an autonomous driving algorithm for a Formula SAE (Society of Automobile Engineers) vehicle. The low-level controller and actuators were setup and studied. The acceleration, braking and steering by wire system equipped in the vehicle were documented and integrated with the low-level controller and safety module [4]. Mokhiamar et al. proposed a control system for skid steering vehicles to enhance the motion dynamics, which especially has a significant benefit on stabilizing the lateral motion and improving the handling characteristics [5]. Gruyer et. al. surveyed recent studies on autonomous driving technologies and controls for the purpose of emulating and replacing the driver’s behaviors with robotic functions. Regarding the autonomous level, the successful beginning steps include some well-known assistance systems such as adaptive cruise control (ACC), automatic emergency braking (AEB), electronic stability control (ESC), lane departure warning (LDW), etc. [6]. In [7], the authors proposed an interacting multiple model approach based on an observer for highly non-linear vehicle dynamics. Through experimental vehicle signals, the algorithm has shown a rapid and robust identification of the faults from the sensor and actuator. For autonomous vehicles, the drive-by-wire technology is regarded as the foundation to increase vehicle control. From various x-by-wire functions, the steer-by-wire system might be the most crucial one in a vehicle. To meet the drivers’ maneuvers and commands, the most significant issue is to handle the wheels. In the research, a proposed sliding mode predictive tracking control (SMPC) has been proposed to deal with the conditions of model uncertainty and disturbance for tracking the wheel steering angle [8]. An integrated system of an electric assisted steering motor and sensors can replace the mechanical steering system in the vehicle. However, two crucial issues should be concerned before commercialization of steer-by-wire systems: (1) maintaining reliability and (2) improving fault-tolerance, where fault detection and isolation (FDI) is required [9]. In [10], Wang et al. studied an autonomous vehicle driving system to achieve such applications. Several key issues including object recognition, classification methods, data registration, data fusion and 3D city modeling were concerned. In complex and changing urban areas, new mobile laser scanning (MLS) systems will be considered for applications. Furthermore, developing rapid, automated and intelligent techniques is required by using machine learning-based methods and a special processing framework [10]. Gao et. al. studied the development of intelligent transportation system (ITS) and vehicle to X technologies capable of detecting a large number of traffic information in the test vehicle [11]. Siegel et al. proposed modern vehicles with an interface for cellular, mesh networking, WiFi, etc. These technologies cover a large variety of range, latency, bandwidth and other specifications as well as market penetration. Moreover, the study revealed that the radio system possessed different robustness to motion, line-of-sight obstruction and antenna design, and thus not all technologies can be properly employed for the specific application [12].

Therefore, this study will focus on the development and application of the go kart industry. From the original steering column, a power steering mechanism capable of manual/remote driving switch control was added. When an abnormal situation occurs, the vehicle control unit (VCU) switches the remote driving mode to manual driving so that the driver can independently control the go kart steering wheel to change the direction of the vehicle. This study includes the design of a steer-by-wire system and signal output of an accelerator with a braking pedal and a VCU that can achieve simple remote driving. The go kart that preserved the original steering wheel, accelerator and braking pedals was further provided with manual driving capable of dual-function mode switching.

## 2. Mechatronics of the Drive-by-Wire

### 2.1. Hardware Configuration of the Acceleration/Brake-by-Wire System

**Figure 1:**
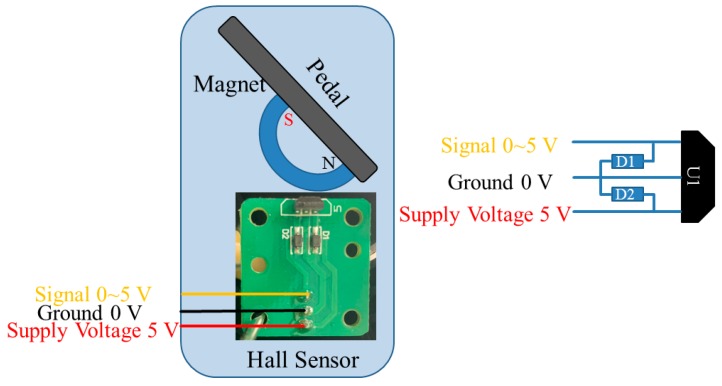
The working principle of the acceleration/brake-by-wire system.

**Figure 2:**
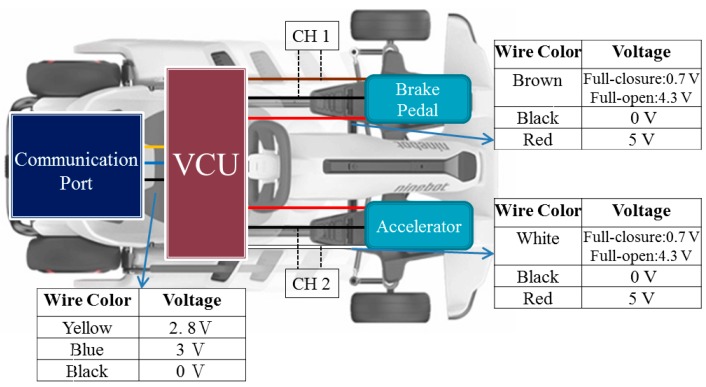
The schematic configuration of the acceleration/brake-by-wire system.

The accelerator and braking pedal were developed using Hall-effect sensors. Based on the principle of voltage, divider circuits for the sensors of the accelerator and braking pedal were regulated to output 0–5 V voltage, as shown in Figure 1. When the sensor position of the accelerator/braking pedal was changed, the voltage was converted into the position signal of the accelerator/braking pedal through the change of the induced voltage. Finally, the accelerator/braking pedal output an output signal to the VCU. The schematic configuration is shown in Figure 2.

**Figure 3:**
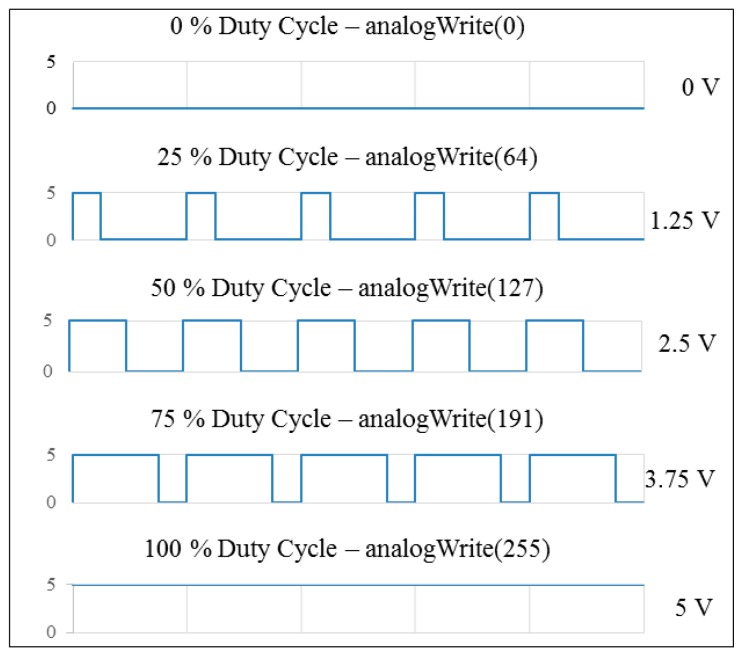
The schematic configuration of the acceleration/brake-by-wire system.

**Figure 4:**
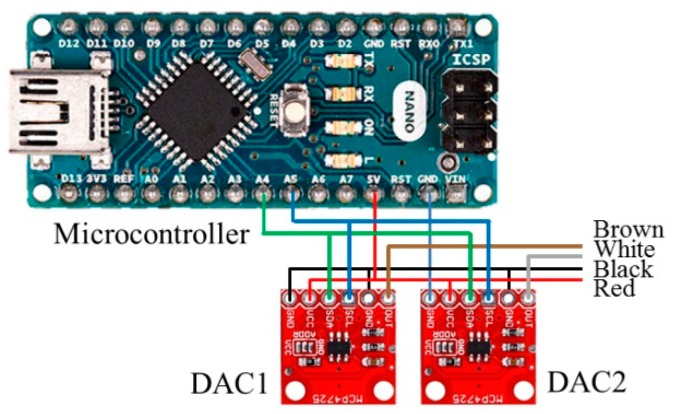
The schematic configuration of the acceleration/brake-by-wire system.

The digital-to-analog converter (DAC) outputs analog voltages from a microcontroller, and the DAC module is an I^2^C (Inter-Integrated Circuit) bus-controlled DAC. A DAC allows the user to send an analog signal, such as a sine wave, from a digital source, such as the I^2^C interface on the microcontroller. The 12-bit DAC converter can accept up to 4096 possible inputs to provide an analog output, where an output value of zero is zero and an output value of 4095 is full scale. The full scale is determined by the reference voltage that supplies the VCC pin. In addition, the supply voltage can be any value within 0–5 V. The microcontroller programming generates PWM (pulse width modulation) values within 0–255; the DAC module transfers the input value in a range of 0–4096 to the output value mapped to 0–5 V form, as shown in Figure 3. The schematic configuration is shown in Figure 4.

### 2.2. Mechatronics of the Steer-by-Wire System

**Figure 5:**
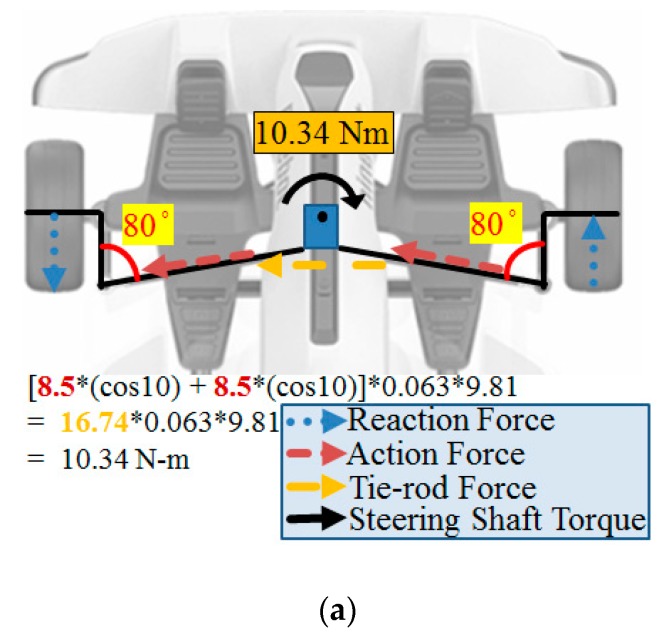

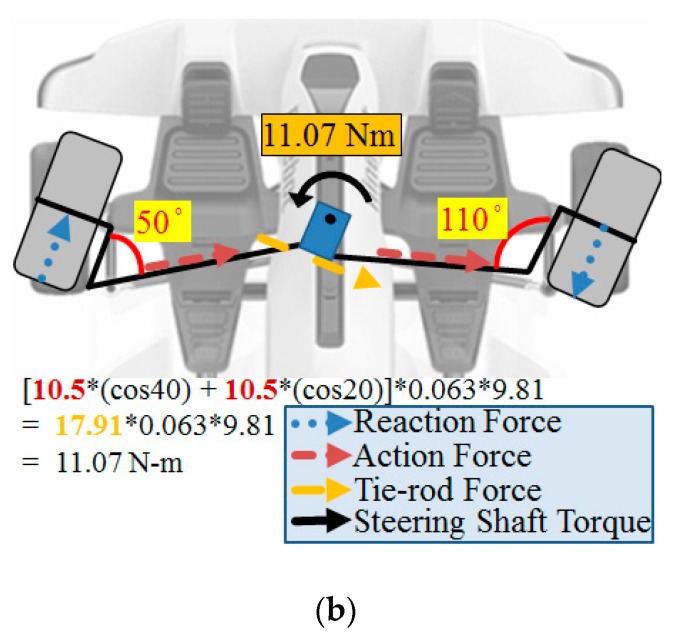
Schematic configuration of the steer-by-wire system by ideal design: (**a**) neutral position; (**b**) extreme position.

The schematic configuration of an ideal steer-by-wire steering system is shown in Figure 5. According to the expected steering requirements from the driver, the output torque of the steering motor is regulated. The 12 V (direct current voltage) is used to supply power to convert the motion of the steering wheel into linear motion. The minimum steering torque is 10.34 Nm, as shown in Figure 5a; when the steering is in the extreme position, the minimum steering torque is 11.07 Nm, as shown in Figure 5b. Thereby, a smooth steering function and desirable feeling of balance and motion stability of exact linearity can be obtained under various driving situations. The driving force and torque changes of the steering shaft are shown in Table 1.

**Figure 6:**
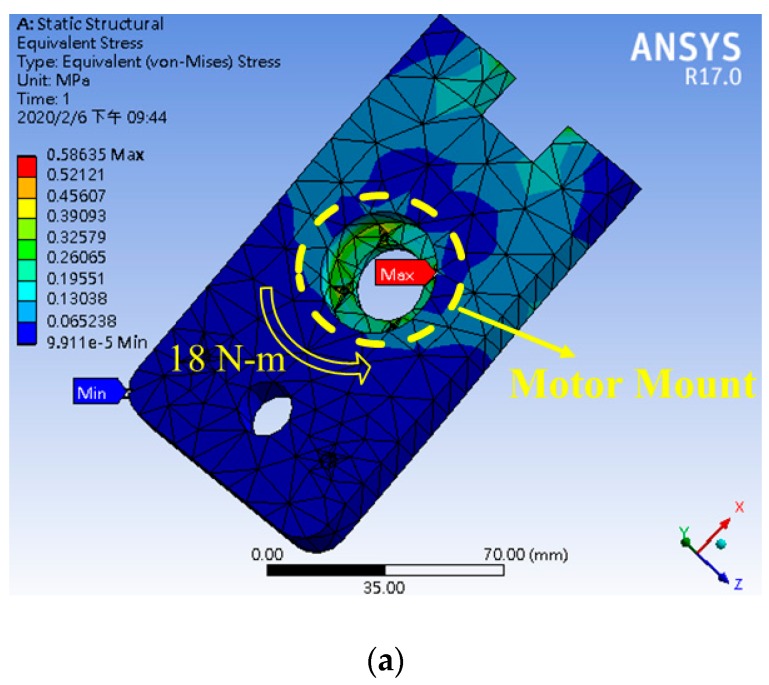

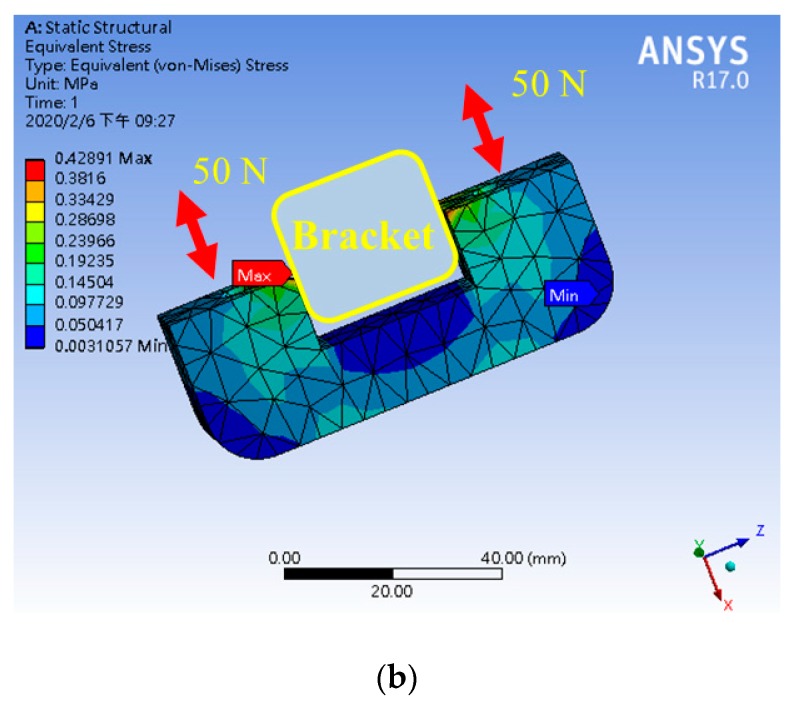
(**a**) Motor mount vs. stress for the steer-by-wire system; (**b**) bracket vs. stress for the steer-by-wire system.

**Figure 7:**
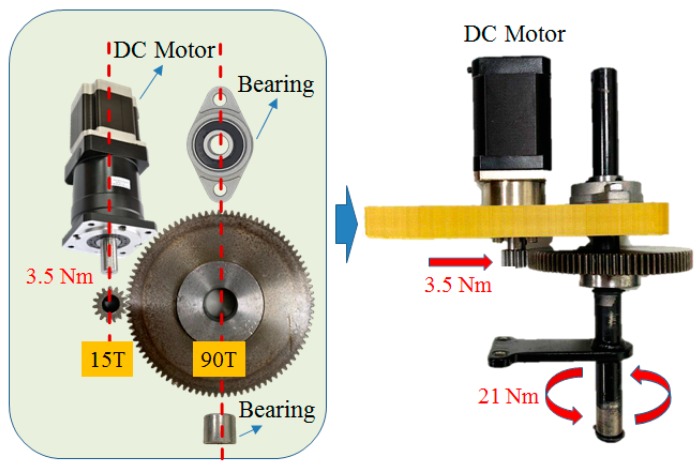
Mechanical design of the steer-by-wire system.

The stabilization function of linear displacement makes it easier for the driver to control the vehicle when driving under the bumpy road. The Creo Parametric® (2.0, PTC Needham, MA, USA) was first used to design the motor mount, and the ANSYS® (R13, ANSYS, Canonsburg, PA, USA) was utilized for stress analysis on the selected 6065 aluminum alloy. The stress analysis results show that the mount is capable of carrying 18 Nm of rotational torque, as shown in Figure 6a. In addition, the mechanism is fixed on the steering bracket of the go kart. The direction of the force is along the contacted surfaces on both sides, and the fixed tensile force of 5 kg is simulated, as shown in Figure 6b. However, the maximum torque and steering torque of the DC (Direct Current) motor is 3.5 Nm and 21 Nm respectively, which meets the requirement of real vehicle steering. At present, the steering system is not equipped with a steering angle sensor and a steering potentiometer. It only outputs the steering torque based on the number of steps of the stepping motor and controls the steering displacement of the steering wheel. The steering angle cannot be a feedback signal because the steering is not exact. The research will correct this problem in future, and the actual architecture is shown in Figure 7.

## 3. Mechatronics of the Vehicle Control Unit

### 3.1. Hardware Configuration of the Remote Controller

**Figure 8:**
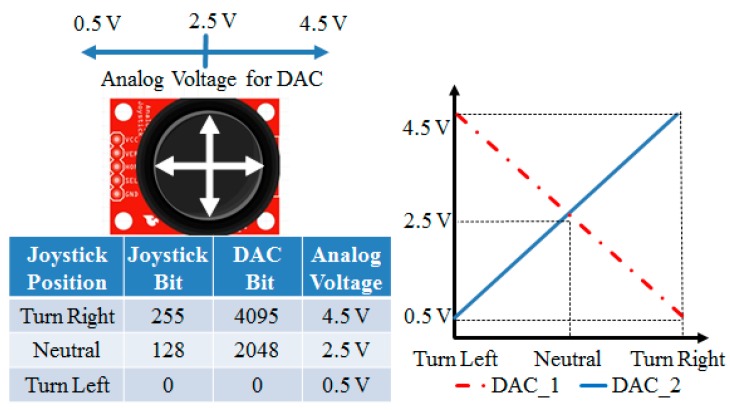
The control configuration of the remote controller.

**Figure 9:**
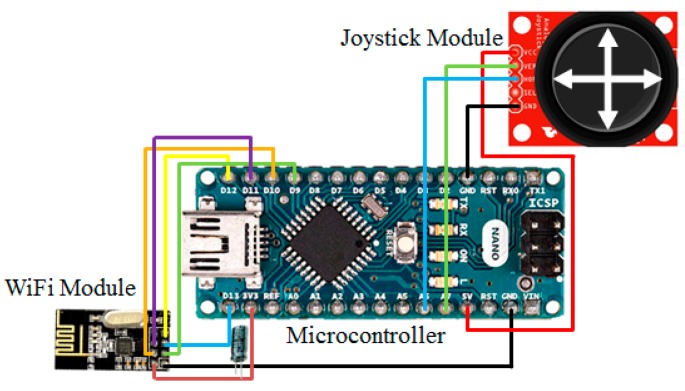
The schematic configuration of the remote controller.

The remote control function is realized using wireless transmission. The channel selection of output power and protocol setting are controlled through the serial peripheral interface (SPI), and connected to various microcontroller chips for completing the transmission work of wireless data, as shown in Figure 8. The schematic configuration reveals that the digital signal of the microcontroller is converted to an analog output of 0.5 to 4.5 V using a DAC module, and a linear change of left-to-right rotation and setting 2.5 V under the center of the steering wheel are defined. The remote control is provided for the VCU to control the positive/negative rotation of the steering motor and steering signals, as shown in Figure 9.

### 3.2. Hardware Configuration of the Vehicle Control Unit

**Figure 10:**
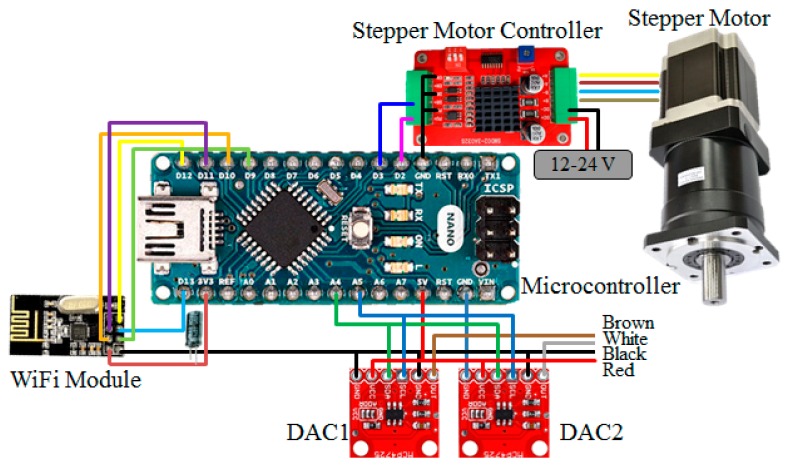
The schematic configuration of the vehicle control unit.

This study integrated the independent controls of the steering wheel, accelerator and brake pedal, as shown in Figure 10. If the status of the manual driving signal is determined, the VCU control strategy cancels the remote control mode of the go kart, and switches back to the manual driving mode. Therefore, the mode selection between the remote driving and the manual driving can be realized, and provides a reaction time for the driver. The schematic configuration of the VCU consists of two DACs, providing independent control of the accelerator and brake pedal, controlling the DC motor through the motor driver to complete independent control of the steering wheel and integrating a wireless transmission receiver, as shown in Figure 10. The indexes of optimization include lower cost, higher performance and better feasibility. The optimization process includes three steps: 1. Building the specification table of the lowest control precision and fast system response time of the acceleration system, brake system and the steering system. 2. Selecting the optimal combination of the acceleration system, brake system and the steering system from the table in step 1. 3. Calculating the appropriate steering motor size and the gear ratio and designs of the micro-controller with the 8-bit control precision.

### 3.3. Control Strategy of the Vehicle Control Unit

**Figure 11:**
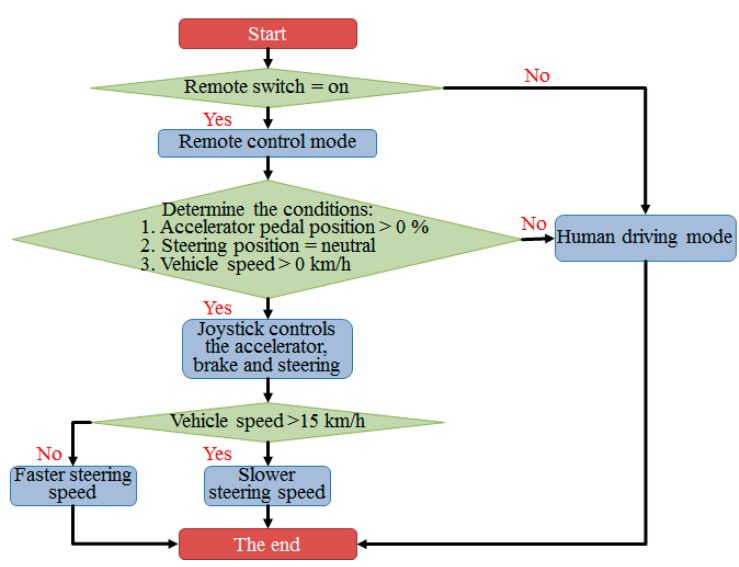
The control flow of the vehicle control unit.

In order to achieve the remote control mode, the control flow is shown in Figure 11. First, a remote control switch is provided on the go kart. When the conditions of the remote control mode are met, the VCU will activate the remote control mode and the vehicle will be controlled by the remote control. Next, if the conditions of the acceleration pedal >0%, the steering wheel position is neutral and the vehicle speed >0% are met, the control of the throttle, brake and steering can be executed by the controller. If the human driving mode is on or the above conditions are not satisfied, the VCU decelerates the vehicle to stop status while the driver takes over the control of the vehicle. As vehicle speed is less than 15 kph with the larger wheel turning angle, the wheel turning angle is decreased while steering or parking; as the speed is higher than 15 kph with the smaller wheel turning angle, the stability of the straight driving can be increased. The control strategies accommodate various driving environments and instantaneous drivers’ requirements.

## 4. Experiment Results

**Figure 12:**
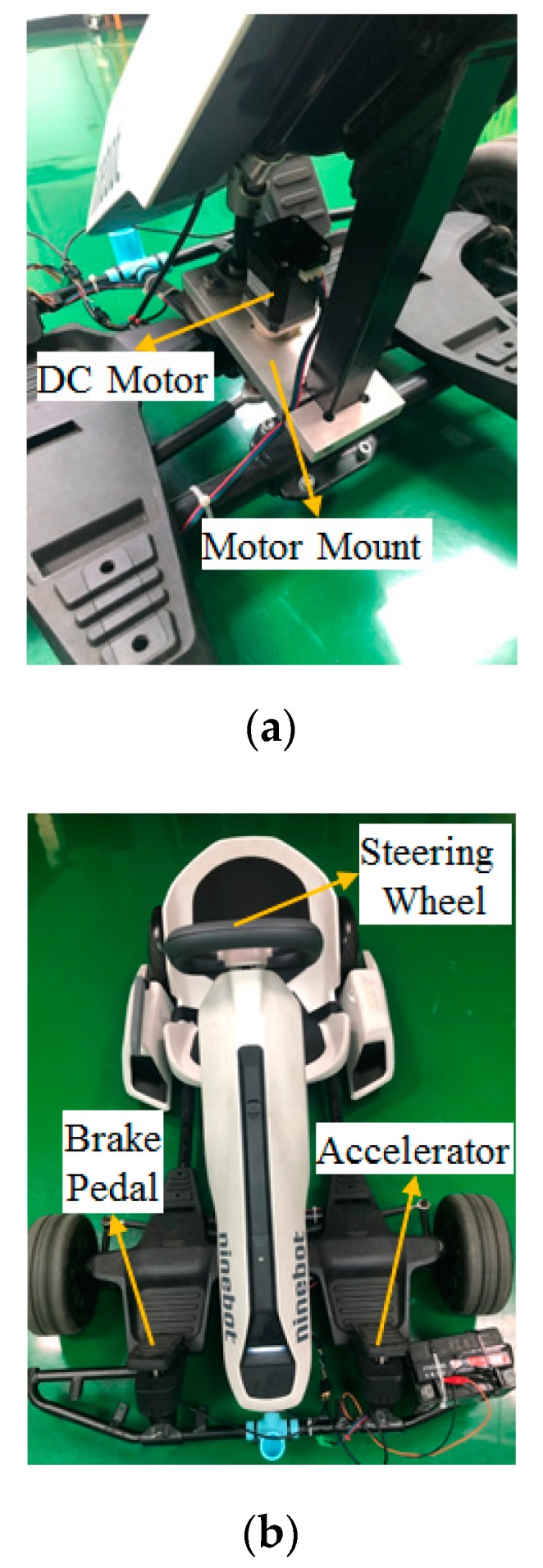
(**a**) The hardware configuration of steer-by-wire in a real go kart; (**b**) hardware configuration of drive-by-wire using the steering wheel, accelerator and brake pedal.

In this study, the drive-by-wire system with a steer-by-wire system for the go kart was built, as shown in Figure 12a. The control-by-wire of the steering wheel integrated with the accelerator and brake pedal steering were developed for the electric go kart. In the future, this will provide the foundation for the development of autonomous driving technology, as shown in Figure 12b.

**Figure 13:**
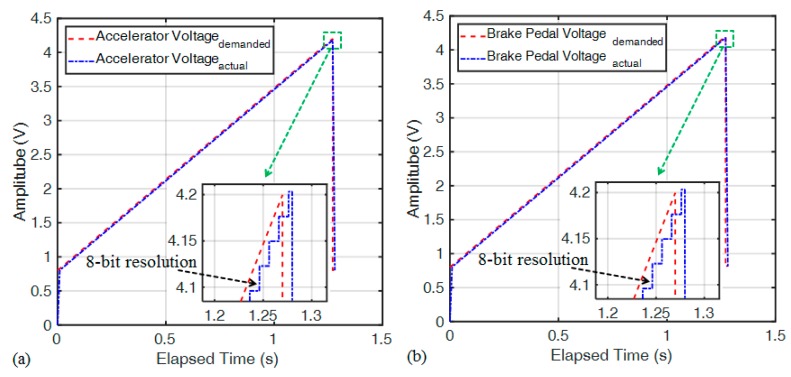
(**a**) Accelerator voltage vs. time for the drive-by-wire; (**b**) brake pedal voltage vs. time for the drive-by-wire.

The accelerator and brake pedal control methods are the same for drive-by-wire. As an example, for the accelerator, the output command resolution of actual control is 8-bit, and the demanded as well as actual delay time is 1 ms, as shown in Figure 13a. In addition, the brake pedal mainly controls the drive motor to reverse for achieving the braking effect. The output command resolution of actual control is 8-bit too, and the demanded as well as actual delay time is 1 ms, as shown in Figure 13b.

**Figure 14:**
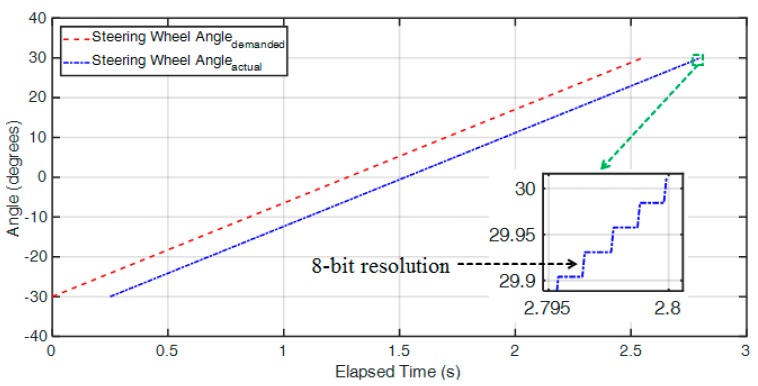
Steering wheel angle vs. time for the drive-by-wire.

The steering wheel angle is treated as the objective of the steering control for drive-by-wire. Here, the output command resolution of actual control is 8-bit, and the demanded as well as actual delay time is 25 ms, which is mainly limited by the physical stepper motor. Attempting to achieve exact position control will slow down the system response, as shown in Figure 14.

**Figure 15:**
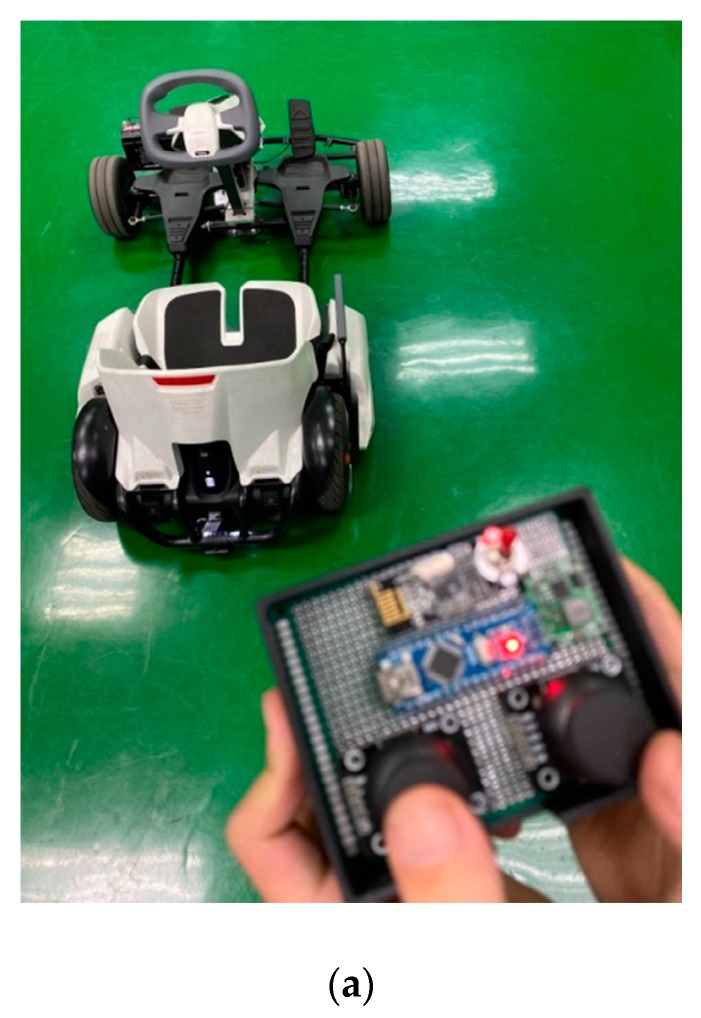

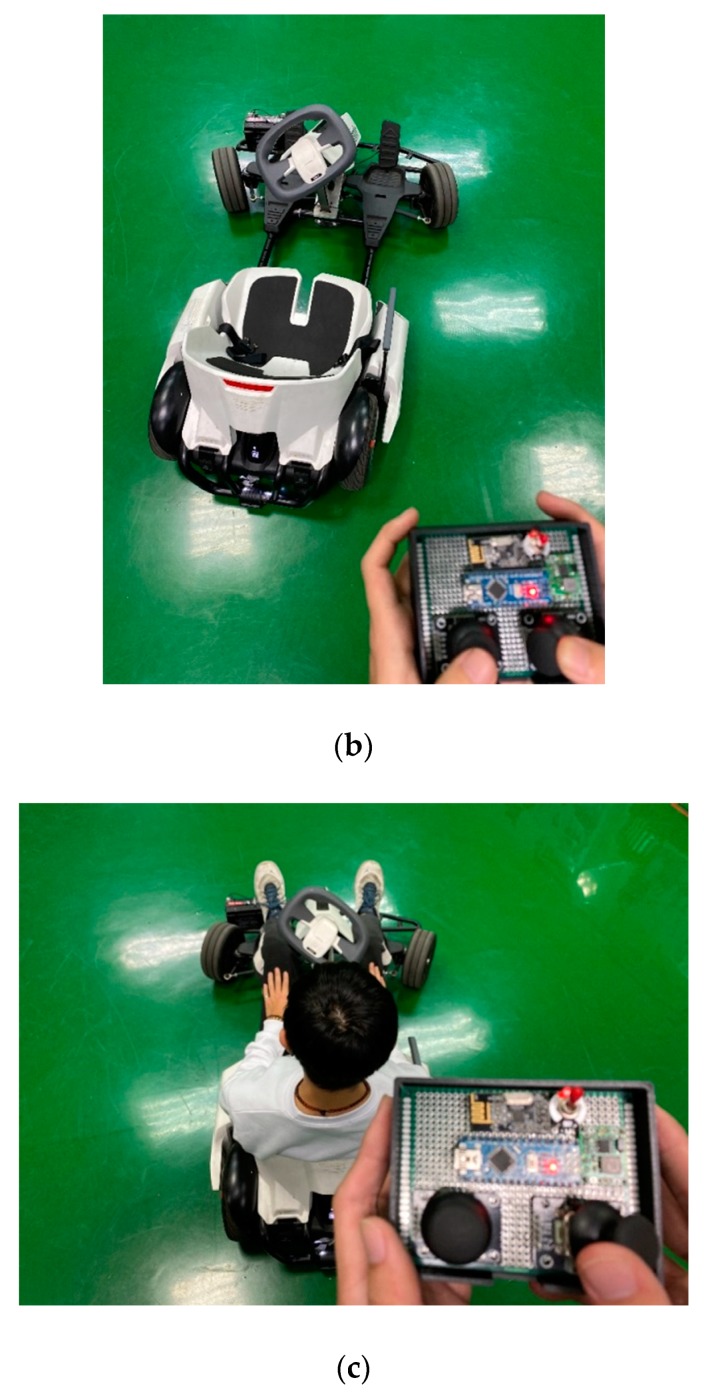
The system verification of drive-by-wire: (**a**) remote control to go straight; (**b**) remote control to turn left; (**c**) remote control to turn right under the passenger mode.

This study was completed using the remote control mode for the drive-by-wire system, and the corresponding specification is shown in Table 2. First, the test was carried out for the remote control mode to drive straight in unmanned mode, as shown in Figure 15a. In the same mode, the test was performed to turn left in unmanned mode, as shown in Figure 15b. Finally, the remote control in passenger mode was tested to verify the feasibility of the wire control system of the electric go kart for meeting the technical development goals, as shown in Figure 15c.

## 5. Conclusions

This paper presents a hardware platform for an electric go kart equipped with a drive-by-wire system possessing a remote control mode to complete the functions of acceleration-by-wire, brake-by-wire and steer-by-wire. The feasibility of applying this to the real vehicle was evaluated for the remote control mode integrated with the drive-by-wire system. The results of the acceleration-by-wire and brake-by-wire experiments showed that the output command resolution of actual control was 8-bit, and the demanded as well as actual delay time was 1 ms; the steer-by-wire experiment results showed that the output command resolution of actual control was 8-bit, and the demanded as well as actual delay time was 25 ms.

This research aimed at the experimental assessment of the x-by-wire system of a light-duty vehicle with extra low cost as well as optimized performance (drivability, safety, operability, robustness). Therefore, the industrial contributions are solid. The benefits and novelty can be summarized as follows:

(1) Competitive cost for aftermarket commercialization: a micro-controller with the 8-bit control precision and a WiFi module are integrated for providing the full function focusing on light-duty vehicles. With the modified steering system, the total cost is USD 200, which is extremely competitive for the aftermarket x-by-wire kits. All light-duty vehicles with peak output power below 2 kW can be equipped.

(2) Self-developed customized VCU: the micro-chip, WiFi module and the DAC interface are properly integrated for the VCU hardware based on the customized requirements of the I/O ports and the wireless data transmission rate. The research can be slightly modified and implemented to other types of vehicles (golf carts, all-terrain vehicles, etc.) efficiently to significantly reduce the R&D period and resources for developing another x-by-wire VCU. For the standard operation procedure (SOP) of the intelligent human/machine interface switch, in order to achieve the remote control mode, the control flow is shown in Figure 11. First, a remote control switch is provided on the go kart. When the conditions of the remote control mode are met, the VCU will activate the remote control mode and the vehicle will be controlled by the remote control. If the conditions of the acceleration pedal >0%, the steering wheel position is neutral and the vehicle speed >0% are met, the control of the throttle, brake and steering can be executed by the controller. If the human driving mode is on or the above conditions are not satisfied, the VCU decelerates the vehicle to stop status while the driver takes over the control of the vehicle.

(3) Standard operation process for the customized x-by-wire system: the drive-by-wire system should concern lower cost, higher performance and better feasibility. The optimization process is as follows: (1) Building the specification table of the lowest control precision and fast system response time of the acceleration system, brake system and the steering system. (2) Selecting the optimal combination of the acceleration system, brake system and the steering system from the table in step 1. (3) Calculating the appropriate steering motor size and the gear ratio and designs of the micro-controller with the 8-bit control precision.

(4) Outstanding performance: for the x-by-wire system, we have improved the low-speed drivability as well as high-speed stability. A larger steering angle of the front wheels in response to a specific rotated angle of the steering wheel has been devised when cornering or parking at low speed, while a smaller one has been designed for high speed. Meanwhile, the system accommodates various driving environments and drivers’ requirements.

## Figures and Tables

**Table 1:** Specification of the steering shaft torque.

Steering Wheel Position	Cargo Weight (kg)	Tie-Rod Force (kg)	Steering Shaft Torque (Nm)
Neutral	70	16.74 (Dual Wheel)	10.34
Extreme	70	17.91 (Dual Wheel)	11.07

**Table 2:** The main specifications of the go kart.

Item			Parameters
Go Kart	Weight	Curb (kg)	≤40
		Gross (kg)	110
Propulsion	Motor	Type Peak power (kW) Peak torque (Nm)	Direct Current 2 70
Drive-by-wire	Acceleration-by-wire	Resolution of actual control (bit) Delay time (ms)	8 1
	Brake-by-wire	Resolution of actual control (bit) Delay time (ms)	8 1
	Steer-by-wire	Resolution of actual control (bit) Delay time (ms)	8 25
